# Supplementary material for: Causal Relationship between Aging and Anorexia Nervosa: A White-Matter-Microstructure-Mediated Mendelian Randomization Analysis
Source: Biomedicines. 2024 Aug 16;12(8):1874. doi: 10.3390/biomedicines12081874 (PMC11351342; doi:10.3390/biomedicines12081874)
Supplement: Supplementary file 1 [file biomedicines-12-01874-s001.zip › Supplementary Figure/Figure S4/Singe SNP plot of MR analysis for TL to ubm-b-1525.pdf]

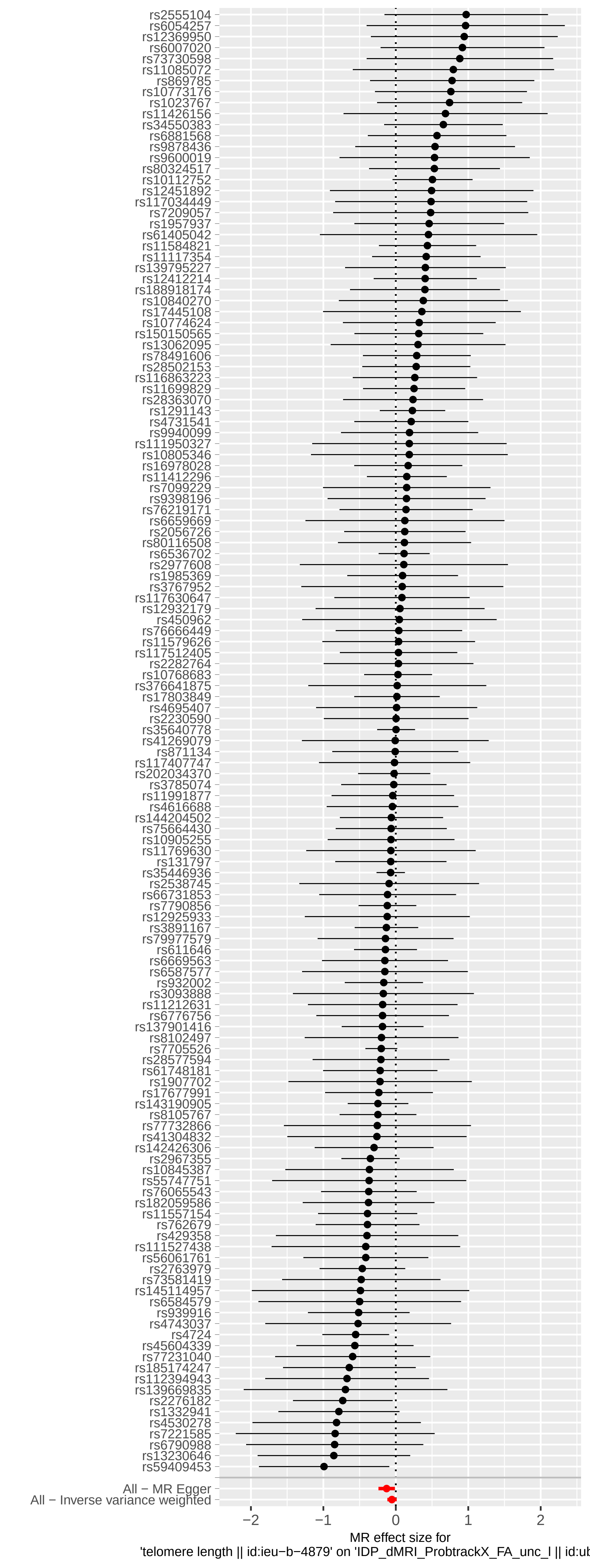
rs2555104
rs6054257
rs12369950
rs6007020
rs73730598
rs11085072
rs869785
rs10773176
rs1023767
rs11426156
rs34550383
rs6881568
rs9878436
rs9600019
rs80324517
rs10112752
rs12451892
rs117034449
rs7209057
rs1957937
rs61405042
rs11584821
rs11117354
rs139795227
rs12412214
rs188918174
rs10840270
rs17445108
rs10774624
rs150150565
rs13062095
rs78491606
rs28502153
rs116863223
rs11699829
rs28363070
rs1291143
rs4731541
rs9940099
rs111950327
rs10805346
rs16978028
rs11412296
rs7099229
rs9398196
rs76219171
rs6659669
rs2056726
rs80116508
rs6536702
rs2977608
rs1985369
rs3767952
rs117630647
rs12932179
rs450962
rs76666449
rs11579626
rs117512405
rs2282764
rs10768683
rs376641875
rs17803849
rs4695407
rs2230590
rs35640778
rs41269079
rs871134
rs117407747
rs202034370
rs3785074
rs11991877
rs4616688
rs144204502
rs75664430
rs10905255
rs11769630
rs131797
rs35446936
rs2538745
rs66731853
rs7790856
rs12925933
rs3891167
rs79977579
rs611646
rs6669563
rs6587577
rs932002
rs3093888
rs11212631
rs6776756
rs137901416
rs8102497
rs7705526
rs28577594
rs61748181
rs1907702
rs17677991
rs143190905
rs8105767
rs77732866
rs41304832
rs142426306
rs2967355
rs10845387
rs55747751
rs76065543
rs182059586
rs11557154
rs762679
rs429358
rs111527438
rs56061761
rs2763979
rs73581419
rs145114957
rs6584579
rs939916
rs4743037
rs4724
rs45604339
rs77231040
rs185174247
rs112394943
rs139669835
rs2276182
rs1332941
rs4530278
rs7221585
rs6790988
rs13230646
rs59409453
All − MR Egger
All − Inverse variance weighted
−2
−1
0
1
2
MR effect size for
'telomere length || id:ieu-b-4879' on 'IDP_dMRI_ProbtrackX_FA_unc_l || id:ub
